# “*To Have a Father, Maybe I Was Going to be a Better Person*”: A Qualitative Study Exploring the Effects of Biological Father Absence on Young Men in South Africa

**DOI:** 10.1007/s43151-025-00181-2

**Published:** 2025-05-30

**Authors:** Campion Zharima, Elton Mboneli, Lerato Tsotetsi, Stefanie Vermaak, Phumla Madi, Busisiwe Nkala-Dlamini, Avy Violari, Rachel Kidman, Amy Hammock, Janan Dietrich

**Affiliations:** 1 Perinatal HIV Research Unit (PHRU), Faculty of Health Sciences, University of the Witwatersrand, Johannesburg, South Africa; 2 African Social Sciences Unit of Research and Evaluation (ASSURE), Faculty of Health Sciences, University of the Witwatersrand, Johannesburg, South Africa; 3 Department of Social Work, Faculty of Humanities, University of the Witwatersrand, Johannesburg, South Africa; 4 Program in Public Health and Department of Family, Population, and Preventive Medicine, State University of New York at Stony Brook, Stony Brook, NY USA; 5 School of Social Welfare and Program in Public Health, Stony Brook University, Stony Brook, NY USA; 6 Health Systems Research Unit, South African Medical Research Council, Bellville, South Africa

**Keywords:** Fatherhood, Adolescence, HIV/AIDS, Youth, South Africa

## Abstract

Biological father absence affects children’s well-being and development, with effects that can persist into adulthood due to associated factors such as economic hardship and psychological distress. In South Africa, where approximately 63% of children are not living with their biological fathers, understanding how this absence affects young people is crucial. This study explores how the absence of biological fathers impacts adolescent boys as they transition into adulthood. The sample for this study was drawn from Tsamaisano, a larger longitudinal study (2020–2023) of 498 young males, some living with perinatal HIV and others who are HIV negative. We focused on 31 participants aged 16–20 who participated in in-depth interviews about their experiences with violence in their households and communities and reported the absence of a biological father in their upbringing. Interviews were recorded, transcribed, and thematically analyzed. Four themes emerged: (1) the absence of biological fathers caused emotional pain, leading some participants to self-isolate and/or engage in aggressive behaviors as coping mechanisms; (2) participants perceived a loss of cultural and masculine identities, feeling disconnected from their paternal heritage; (3) they described experiencing poor financial security and limited emotional support, with mothers and other family members sometimes filling in the gaps; (4) despite these challenges, some participants reached stages of acceptance and closure over time, often supported by extended family members providing care. This study highlights the need for interventions targeting adolescent boys that address their emotional, social, and developmental needs, factors that are vital for their overall health and have lasting implications for their adult lives.

## Introduction

Fathers play a critical role in the development and well-being of their children (Hornstra et al. 2022). Studies show that children raised without present biological fathers tend to experience poorer health, well-being, behavioral outcomes, and socio-economic status compared to those whose fathers are present and involved (Freeks 2017; TenEyck et al. 2023). Research in South Africa also underscores the link between father absence and adverse outcomes in education, mental health, and early initiation of substance use among children (Kim et al. 2021; Salami & Okeke 2018). Moreover, boys whose fathers are absent, are more likely to engage in risky sexual behavior, such as unprotected sex or early sexual debut, and violent behavior (Kim et al. 2021).

While extensive work has explored father absence and the reported importance of fathers in children’s lives, many children in South Africa still grow up without their biological fathers (Teagle n.d.; van den Berg et al. 2021). Moreover, although conventional measures of father absence (such as co-residence) are useful, they sometimes fail to capture the complex dynamics of paternal involvement and its impact on children’s well-being beyond reported causes and outcomes (Liu et al. 2023; Xiang & Zhou 2023). This limitation can result in oversimplified or inaccurate narratives about fatherhood in South Africa, highlighting the need to examine how adolescents themselves perceive and navigate father absence. This is especially relevant for young men in the South African context, where multiple intersecting forces shape their lives. Examining these issues can help inform interventions that not only address immediate concerns but also support healthier transitions into adulthood.

## Literature Review

Fathers can play multiple roles in their children’s lives—as primary parents or co-parents, whether resident or non-resident (Yogman & Eppel 2022) and with varying levels of involvement that may change over time and place (Epstein 2010). This makes father absence a complex phenomenon that is often difficult to measure. Despite the lack of definitional clarity in the literature, father absence encompasses a range of situations, including involuntary causes such as death and work commitments, and voluntary factors such as negligence or disinterest (East et al. 2006). In some cases, absence is perceived as a lack of emotional support, communication, caregiving, or financial assistance to the child (Coley & Medeiros 2007). These varying circumstances can impact adolescents in distinct ways, and children may have to navigate situations in which a father’s presence is not easily understood. Due to this complexity, some researchers have attempted to categorize father absence into broad types: “absent and unknown,” “absent but known,” “absent and deceased,” and “unknown and deceased” (Padi et al. 2014).

The complex web of factors influencing father absence can be context-specific and include cultural, social, political, and historical influences (Malinga & Ratele 2022; Padi et al. 2014), as well as parenting styles (Diniz et al. 2023; Garcia et al. 2022; Seya et al. 2023). In this paper, we focus on father absence in South Africa. Over the past 50 years, South African fathers have increasingly assumed caregiving responsibilities in addition to their traditional breadwinning roles, despite the persistent influence of gender role stereotypes (Malinga & Ratele 2022). The State of South Africa’s Fathers report revealed that a significant proportion of fathers actively participate in various aspects of their children’s lives, including providing financial support, engaging in outdoor activities, and assisting with homework (van den Berg et al. 2021). The report further showed that 63% of fathers provided financial support, 66% helped with homework, and 58% took time to play with their children outside (van den Berg et al. 2021).

Some scholars have focused on the role played by social fathers as alternative male figures who fill the gap when biological fathers are absent (Kesebonye & Amone-P’Olak 2021). Thus, while biological father absence may be high in many African families, the role of social fathers is often overlooked in strengthening links to the family lineage and community (Nathane & Khunou 2021). Social fathers, especially when the role is assumed by a close relative, can bring emotional support and connection, model positive masculinity, and preserve the cultural identity for young men (Nathane & Khunou 2021). However, social fathers can often bring new challenges; for example, studies have shown an association between the presence of unrelated adult males and a higher risk of child maltreatment (Berger et al. 2009). In low-income families, social fathers may complicate family structures if they come with children from previous families, as resource constraints make it harder to provide for a bigger family (D’Andrade & Sorkhabi 2016).

Father absence in South Africa can be understood as a result of the historical impact of apartheid (Ratele et al. 2012). In this system of racial segregation, Black men were forced to work in distant mines away from their homes and consequently spent little time with their families. The results can still be felt today: African fathers’ precarious employment can hamper their involvement in their children’s lives, as the necessity to provide financially limits their physical presence (Malinga & Ratele 2022). Moreover, the history of South Africa is marked with violence, which has carried over to the post-apartheid society with homicides, suicides, incarcerations and crime affecting families (Jewkes & Morrell 2018). The 2019 general household survey in South Africa revealed that 63% of children do not live with their biological fathers, despite the fact that most fathers are still alive (87%), with 11% deceased or the status of 2% unknown (Mavungu 2013; van den Berg et al. 2021). Finally, the socio-economic challenges presented by high unemployment and poverty in South Africa intersect with notions of masculinity and fatherhood to exacerbate these challenges (Cochran et al. 2023; Patel & Mavungu 2016). Some research has highlighted the challenges of split families and distant parenting due to their often-unmitigated ramifications on children while scrutinizing the effects of traditional gender roles on parenting (Seepamore 2016).

Another key factor shaping father absence in South Africa has been the HIV epidemic (Meintjes et al. 2010). In the sub-Saharan African region, South Africa has the third highest number of HIV-related deaths, with 720,000 children under the age of 15 orphaned due to AIDS (Bradshaw et al. 2016; Mojola et al. 2022; UNAIDS 2023). Children orphaned due to HIV/AIDS are more likely to report higher rates of emotional, behavioral, and physical problems than those who are orphaned by other causes (Breckenridge et al. 2019). Research on HIV orphans indicates that they often receive low levels of psychosocial support, leading to poor educational, behavioral, and psychological outcomes, particularly when living with unaffectionate caretakers (Verma & Lata 2015). Even as extended family members step in as caregivers, their work demands in the context of widespread poverty often reduce the time they can spend with children (Kidman et al. 2007).

Despite extensive research on the causes and consequences of father absence, there is a notable lack of research capturing the perspectives of young people growing up without their biological fathers (Malherbe & Kaminer 2022). Given the well-documented health and behavioral consequences of father absence, it is important to explore how father absence affects young people during adolescence, a pivotal period of transition into adulthood, marked by questions of identity, belonging, and self-esteem. This is especially relevant in the context of South Africa, where rates of father absence are notably high. The aim of this paper is to explore how the absence of biological fathers affects adolescent boys as they transition into adulthood.

## Methods

### Study Setting

The study was conducted at the Perinatal HIV Research Unit (PHRU), located at Chris Hani Baragwanath Academic Hospital in Soweto, South Africa. Soweto is a densely populated peri-urban area in the southwest of Johannesburg. The area was a product of colonial apartheid policies designed to segregate Black Africans from the white population (Phillips 2014). Over time, Soweto developed into a predominantly low-income community, characterized by high unemployment, informal settlements, and elevated crime rates—factors that serve as social stressors impacting the livelihoods of its residents (Gilbert & Soskolne 2003).

### Sampling and Recruitment

The main study recruited 498 adolescents living with and without HIV to investigate the effect of violence on adolescent health. The study design included a quantitative and a qualitative component. At the end of the quantitative follow-up, criterion-based sampling was used to select and recruit 52 participants for the in-depth interviews. Participants were categorized based on lifetime experiences of intimate partner violence (IPV; emotional, physical, or sexual), adverse childhood experiences (ACE; low, medium, or high), HIV status (perinatally acquired HIV, no HIV infection), and age (< 18 years or18 years and older). These criteria formed 24 distinct sub-categories to ensure representation across these characteristics. All participants signed a separate informed consent form for their participation in in-depth interviews. The main study exclusively recruited adolescent boys. From the 52 participants in the qualitative component, this paper focuses specifically on the lived experiences of the 31 participants who reported growing up without their biological fathers during the interviews.

We define father absence as a situation where a biologically related male adult lives separately from their child, leading to minimal or no involvement in caregiving, regardless of the underlying reasons. We recognize that there are diverse reasons for biological father absence, and their physical absence from the household does not necessarily equate to non-participation in a child’s development (Chauke & Khunou 2014). However, it can still affect their ability to fulfill their parental roles (Coley & Medeiros 2007) and is thus captured under our definition.

### Study Procedures

In-depth interviews were conducted by a team of four research assistants, comprising three males and one female, all experienced in conducting in-depth interviews and fluent in local languages. Prior to the interviews, research assistants underwent additional training to enhance their interviewing skills. Interviews took place in a secure room at the PHRU and were recorded with durations ranging between 32 and 95 min. For quality assurance, senior researchers regularly reviewed audio recordings and transcripts of the interviews. The South African-based research team convened weekly meetings, while the US-based team joined bi-weekly to discuss challenging interviews and emerging themes. Questions growing out of emerging themes were incorporated into the interview guide to improve it for subsequent interviews.

### Data Collection

Trained interviewers used a semi-structured interview guide that included the following topics: experiences of IPV in relationships, violence in the family, household, and communities, and sexual behaviors. The theme of the absent father emerged as participants spoke about missing parents or caregivers and reactions to missing family members.

### Data Analysis Approach

All audio recordings were transcribed verbatim, translated into English, and verified by the research assistants. Following the completion of all interviews, we developed a coding framework with input from three senior researchers, which was continuously revised to accommodate newer emerging themes. Coding was done using NVivo, a qualitative data analysis software. To ensure reliability, a subset of transcripts was randomly selected for coding verification by three senior researchers for further review.

From the data, we extracted and analyzed quotes that were coded under “Absence of Family Members” or “Reaction or feelings to missing a parent or caregiver” to identify participants with absent fathers and their shared experiences. This resulted in quotes from a sample of 31 participants for analysis. The quotes were clustered into themes following the six-step thematic analysis method outlined by Braun and Clarke (Braun & Clarke 2006). All quotes were grouped, and preliminary labels were assigned based on their semantic content. Analysis of latent meaning was employed, delving beyond surface-level interpretations to uncover deeper meanings within the quotes and themes.

### Ethical Considerations

The study obtained approvals from ethical review committees from [blinded for peer review]. Prior to any study procedures, all participants signed informed consent or assent forms, and parents or caregivers signed consent forms on behalf of participants under 18 years old. Participants also signed a separate consent or assent form for being audio recorded during the in-depth interviews. A counselor was available for those who needed psychosocial support after participation in the interviews.

## Results

The results in this study are analyzed from 31 participants aged 16 to 20 years who reported their biological fathers being absent at the time of this data collection. The sociodemographic characteristics of the sample are shown in Table 1.

**Table 1:** Sociodemographic characteristics of the study sample (*N* = 31)

Characteristics	Details
Age	- Participants ranged in age from 16 to 20 years, with an average age of 17.8 years
HIV status	- 16 participants (52%) were uninfected with HIV (HIV-negative) - 15 participants (48%) were living with HIV (HIV-positive)
Participant education	- 19 participants (61%) were enrolled in school - 12 participants (39%) were not enrolled in school
Biological father’s status	- 17 participants (55%) reported that their biological father is deceased - 14 participants (45%) reported that their biological father is alive
Biological fathers living in the same household	- For 28 participants (90%), their biological father does not live in the household - For 2 participants (6%), their biological father lives in the household - 1 participant (3%) had missing data
Participants’ time living in the household	- 24 participants (77%) have lived in their households continuously since birth - 4 participants (13%) lived in their households for more than 5 years - 2 participants (6%) lived in their households for 1–4 years - 1 participant (3%) had missing data

Among the sample, reasons for father absence varied and included death, incarceration, remarriage or living with another family, unknown whereabouts, or simply not sharing the same household. In addition, most participants reported having poor relationships with their fathers. Participants’ experiences with their fathers’ absence were categorized into the following four key themes: (1) emotional pain and coping, (2) perceived loss of masculine identity and self-worth, (3) strained financial and psychosocial support, and (4) acceptance and closure.

### Theme 1: Emotional Pain and Coping

This theme explores the emotional impact of biological father absence on young men and their coping strategies. Some participants externalized their emotions through anger and aggression, while others adopted strategies such as self-isolation and private grieving. Notably, others demonstrated resilience by avoiding negative coping mechanisms.

Participants reported a range of emotions in reaction to their fathers’ absence, which showed the depth of their pain. One participant recalled the trauma he endured when he was younger:

“*He would come sometimes and go again. It really traumatized me a lot. But now I have a good understanding that he is always busy… but when I was still a child, I didn’t understand. He sat me down and addressed me about his life... just recently*” (IDI_50, N, 18 years).

Similarly, another participant shared the following about the pain he experienced growing up without his father:

“*That one pains me because of my upbringing, since I finished studying, growing up was something else. I even dream of the guy [father]. It’s very painful, it hurts me. Sometimes it doesn’t go away, he doesn’t stay far from my neighborhood, from my house*. *So, lately I bump into him, but we don’t talk, he just looks at me*.” (IDI_44, N, 20 years).

Participants reported various ways of coping with this pain. One way was to retreat inward and self-isolate in order to grapple with feelings of loneliness and longing for parental connection. The following participant describes the grieving he goes through and the emotional void he feels when he is alone:

“*It hurts when you hear other kids saying ‘my Mom or my Dad’ in the streets. It still hurts, but not like before. If I want to cry, I sit alone*” (IDI_03, P, 16 years).

Another participant described a similar reaction to the pain caused by his father’s absence; he internalized his emotions rather than sharing them with others:

“*It has affected me in many ways, when I was growing up friends would talk about their fathers, I’m like where is he. Yes, I did feel that I am not ok, but I noticed it doesn’t help. If something hurts me, it’s better it hurts me alone, I don’t share things. I spoke to no one. It’s just that some things I don’t like to talk about to people*” (IDI_10, P, 18 years).

Processing these painful emotions internally was also a coping mechanism used by another participant whose father had passed away. While he appreciated hearing others speak about his father, their comments also made him feel alone. He describes his longing for him:

“*So, growing up without a father, you hear things in your community that your father loved you. Even at home they tell you your father loved you, or he would do anything for you. But this makes you think of a lot of things...You think about things you could have had, things he would have done for you that you didn’t get*” (IDI_22, P, 20 years).

In contrast to coping by internalizing feelings, some participants reported developing externalized behavioral problems. One participant attributed his short temper to the death of his father:

“*I think it started when my father passed on, that’s when I started being short tempered. Because I had already told myself that he’s one person that will always be there for me in life. So, from his passing on, I started being very short tempered*” (IDI_52, N, 18 years).

Another participant describes how his father’s absence led him to develop quick rage and anger, which can sometimes result in violent behavior:

“*When they provoke me I am very fast. I beat too much, and no one who will stop me. Only the police can stop my rage. It is not as if I make myself appear dangerous, but it is the way I am, when I feel like I can hurt someone and get arrested. So by being alone, I avoid lots of things you see. I don’t want to be around him [father]. I don’t want him, he must stay far away from me. Sometimes I just get angry without knowing the reason.*” (IDI_40, P, 20 years).

The participant acknowledges that his anger stems from resentment toward his father and admits that there are times he does not know where the anger comes from, suggesting a loss of control.

While some participants struggled with managing their emotions, others found comfort in avoiding harmful coping mechanisms, as one participant shares:

“*I knew my father but I didn’t know why he was not coming to check us, he knew that am here but I was always expecting him maybe he will come one day. But it didn’t affect me that much but just a little, because it didn’t lead me to drugs smoking drugs*” (IDI_39, N, 20 years).

### Theme 2: Perceived Loss of Masculine Identity and Self-worth

This theme discusses the impact of father absence on young men’s masculine identities and self-worth. Specifically, participants perceived that their fathers’ absence disconnected them from their personal heritage, spiritual connection, and cultural knowledge, leading to feelings of incompleteness. In some cases, they attributed their life struggles and uncertainties to the absence of their biological fathers.

Father absence affected young men’s masculine identities and self-worth as they transitioned into adulthood. Several participants expressed feeling incomplete because of their disconnection from their paternal families. One participant points out how his father’s absence meant him losing a figure to teach him about his culture, an essential part in his transition into manhood:

“*I needed that daddy figure you see. To have a father or something like that, maybe I was going to be a better person. To teach me culture, I don’t know the culture. In my culture, if you don’t have the father’s side, you are weak. Not really weak but you are not man enough.... When I’m like this I’m not full because my father did not do the ceremony to introduce me, so they don’t know me from his side*” (IDI_39, N, 20 years).

This participant further stated how the disconnect that he feels because of not being in contact with his father and paternal relatives signifies a loss of spiritual protection and luck from his paternal ancestors:

“*I’m a man, but in my culture, I’m not enough. Most Africans we have ancestors from both sides of parents who have to know you. So if they know you on one side only then you are not protected enough and your luck may be on the other side*…. *Even me maybe I don’t know where my lucky are. Because lately it’s the up and down you see in my life. They didn’t abandon me but it’s my father fault that some things are happening*” (IDI_39, N, 20 years).

This participant explains that his father’s absence resulted in the loss of his cultural knowledge, an essential part of his identity. He also attributes many of his life’s struggles and uncertainties to the absence of his father.

Similarly, another participant, whose father lives with another family, felt neglected because his father prioritized his other children while keeping him a secret:

*“The father who gave birth to me does not care about me. I don’t know why. To his family I was a secret. It affected me how he loves his other kids but not me. He is still alive even now but he doesn’t care about me. We don’t talk to each other…I also even forgot that I have a father.”* (IDI_40, P, 19 years).

In this case, the disconnection, both physical and emotional, may have affected his self-worth as the participant perceives his father’s prioritization of his other children over him. In addition to feeling hidden and unacknowledged, the participant expressed how this was worsened by the delayed disclosure of his illness, highlighting the intersectional challenges of father absence and HIV:

*“They hid from me the fact that I am sick, my mother hid it from me. They only told me when I was clever [old] enough. And that affected me even worse.”* (IDI_40, P, 19 years)**.**

### Theme 3: Strained Financial and Psychosocial Support

This theme examines the financial and psychosocial strain caused by the absence of a father, showing the burden of care left behind. Participants shared experiences of being excluded by family members, especially from the paternal family, and their mixed experiences with stepparents who were sometimes unwelcoming.

Participants shared how father absence strained their financial and psychosocial support, often when the burden of care was transferred to other caregivers. One participant reflected on this issue, describing how his unemployed mother struggled to make ends meet after his father’s death:

“*I was asking myself how my father can pass away, and I didn’t even know him. It felt normal because he never even supported me while growing up, but he did bring me to this world, and I do not blame him...I was brought up by my mother and she never had a job and was always struggling so she didn't know how to help me or assist me in some certain things*.” (IDI_49, N, 18 years).

In a unique case, one participant, whose parents are both absent but alive, explains how this forced him to take on adult responsibilities at a young age, as he had to fend for himself:

“*And they [parents] are both useless. I was supposed to be like other children. I was supposed to be normal, I’m not a normal kid. You can see I’m old. I don’t chill with other kids. They make me an old person when I’m still young. It’s not that they are useless but they’re not useful to the things that I need.…Otherwise, I can do my things and live my life and live without them*.” (IDI_32, N, 18 years).

In other cases, family members avoided taking over the caregiving role to evade the associated responsibilities. One participant described being excluded by his father’s family, who presumably assumed that he would seek financial support from them since his father’s passing:

“*I think by not giving me the time, they think if I come back, I will want other things. That is why I’m not interested in them that much. Until I have my own capital then I can go. Even on my father’s funeral, I didn’t go, they didn’t call me so there is nothing that I know there*.” (IDI_13, P, 18 years).

Thus, the participant’s refusal to visit them until he has his own money suggests that he may want to be acknowledged in other ways than as someone seeking financial support.

Father absence also strained psychosocial support and increased the caregiving burden on those who remained, often the mothers. One participant expressed how his mother assumed this responsibility:

“*I look up to my mom because... she is my mother and my father at the same time. I didn’t grow up with my father so.... Like I knew my father but he was not coming to check us, even though he knew that I’m here*.” (IDI_39, N, 19 years).

Another participant, living with HIV, shares how his mother had to oversee his welfare and treatment adherence in his father’s absence:

“*Most of my problems I blame my father. My mother is with me and cares about me, if I take my pills, if I go to fetch my pills, my viral load, if I eat, and how they treat me. He never asked me if I drink my pills or how I am, how the pills treat me or if they cause me headache or loss of appetite, he never asked that.*” (IDI_15, P, 16 years).

While mothers primarily took over the duties of care in the absence of fathers, in some cases, this role was assumed by other family members, who stepped in to provide both financial and social support:

“*My uncle is the one that came and took me to stay with him and took care of me where he could, but then...when there is no money, there's nothing we can do, I had to understand the situation in the house*.” (IDI_49, N, 18 years).

While family members often played critical roles in mitigating the impact of the strains endured, this was not always the case. In some instances where the fathers had passed away, family members sometimes rejected them, as one participant shared:

“*I also had an uncle, who was supposed to raise me up [but didn’t]. I still hold on to the fact that my father is the one that got a job for him, and those are the kind of things that still hurt me till this day.*” (IDI_37, N, 19 years).

When a father is already absent, relying solely on the mother leaves them vulnerable, as her death could leave them unprotected. One participant shared how family members did not support them despite promising to do so:

“*A lot of people were close to my mom and after the funeral they said they would help, but I guess they were just sympathizing with us*.” (IDI_42, N, 17 years).

Father absence also strained psychosocial support systems through its disruption of the dynamics and structure of the family. One participant describes how this affected his communication with parents:

*“Having two parents not living together makes it seem like it is something really serious and hectic you know. Somehow likes it reduces the level of communication to both your parents you know.*” (IDI_27, N, 16 years).

Another participant shares how physical separation negatively affected social support from family members:

“*I am my mother’s only child but my father has five. We are boys only and I’m the middle one. So on my father’s side they are not involved, the other four are staying with my father. I am the only one who stays at my mother’s. I last saw them when I was five.*” (IDI_13, P, 18 years).

Family dynamics and structures were altered when parents started dating or remarried other people. While some of these new adults were supportive, others were unsupportive, sometimes worsening their living arrangements:

“*My biological father died when I was young. I didn’t know him...and my mother was staying with her new man, we are using his surname now. He raised us, me and my siblings but he died in 2016. That’s when she met the current one, and when things changed now everything is not right*.” (IDI_30, N, 16 years).

Some participants shared that their relationships with their absent fathers became even more tenuous when their fathers married other women. One participant, whose mother had passed away, described his experience with a stepmother:

“*It makes me feel like I do not have a father. I feel like my father passed away too you see. Even though I make time to go see him, and he stays with my stepmom, I don’t like her because I saw that she doesn’t like me too, but I don’t want to show her*.” (IDI_42, N, 17 years).

Similarly, another participant described how he feels each time he encounters his father and stepmother:

“*I could say, my stepmother, his wife, is also the one that I see. What pains me is that every, every time when I, when I meet them, it can be like on the street or at a mall, they always look at me, and their look, it like it says something else*.” (IDI_44, N, 19 years).

### Theme 4: Acceptance and Closure

In this theme, participants share their processes of finding acceptance and closure despite the challenges of father absence. Some gradually accepted their fathers’ absence, acknowledging the seemingly unchangeable situations, such as death. Others found closure through support from extended family members, with some maintaining occasional contact with their absent fathers.

Despite the many challenges that participants faced in dealing with the absence of their fathers, some found acceptance and closure, demonstrating resilience over time. Several participants described gradually coming to terms with their fathers’ absence, as one shares:

“*I didn’t really think about it in most times. I am fine now. I am grown up now and understand that it happened, and it is all in the past*.” (IDI_36, N, 19 years).

For others whose fathers died, this came with the realization that they could not change circumstances, as two participants shared:

“*I would say it was hard going through life…It was bad. Like sometimes I wish I had a dad but still there is nothing I can do*.” (IDI_14, P, 17 years).

“*At first when I was young and still growing, it affected me. Even now it still affects me but not like before. But as time went on, I ended up understanding that it was meant to be*.” (IDI_22, P, 19 years).

In each of the above cases, this process of acceptance was gradual, a lessening of emotional pain.

Another participant shared that part of his strength and resilience comes from his belief that his late father is watching over him as he aims to live independently:

“*I’m strong it doesn’t affect me that much because I trust myself and I trust that my father is watching over me. I love myself a lot more than other family members, I like to put myself first that is why I’d like to go out and rent a room*.” (IDI_13, P, 18 years).

Two participants shared how their closure was facilitated by family members, specifically grandmothers, who bridged the gap of absence and brought closure:

“*I got closure from my grandmother because my older brother the one we share a mother he was still at school*.” (IDI_19, N, 17 years).

“*Like I was feeling like an outsider from my family but grandmother showed me love and that there are people that still love me as I love her too*.” (IDI_03, P, 16 years).

However, some participants could not find a similar closure due to the circumstances surrounding their fathers’ absence, such as undisclosed illness or death:

“*I used to sit and not think about him maybe for a year or 6 months then I will think of him and whether he is still alive or how is he doing. Until 2020, that is where I found out that he was sick and can’t walk*.” (IDI_39, N, 19 years).

“*There is a lot of stories so…there’s a lot regarding this…there’s many stories. He was a hustler. He died at the hospital. They said he got sick.*” (IDI_22, P, 19 years).

Participants who had regular communication or contact with their biological fathers or their paternal families described a more positive outlook on life, despite their absence. For example, one participant felt good about his relationship with his father because he was able to talk to him and see him in person:

“*It [the relationship] is fine, it’s fine. Yes, we do talk, like I recently just visited him where he stays*.” (IDI_23, P, 19 years)

Communication and regular contact helped to maintain a sense of connection and normalcy. Another participant whose parents separated shared how keeping in touch with his father’s family helped him cope with his absence:

“*We are alright, no one is angry at another we all good. They [father’s family] have their own house they don’t stay with us…but we do visit them sometimes*.” (IDI_06, P, 19 years).

Similarly, another participant whose parents separated described how communication with his father helped him to come to terms with it:

“*Since I was young, I didn’t understand the situation [separation] well. Obviously it’s good, as long as we’re still talking to each other. It’s good, like he never left us*.” (IDI_43, N, 16 years).

## Discussion

Our study highlights critical unmet needs arising from biological father absence. It further demonstrates that the impact of such absence can be emotional, financial, physical, and/or cultural. For example, father absence had significant emotional effects on young men. Participants exhibited various emotional coping strategies, ranging from adaptation to rebelling. In some cases, not understanding why their fathers were absent led to maladaptive behavior, which manifested as isolation, private grieving, anger, and aggressive behavior. However, some participants managed to avoid harmful coping mechanisms, which could be attributed to their resilience or the presence of supportive social networks.

Existing literature shows that biological father absence can lead to adverse emotional reactions in adolescence and young adulthood, with coping mechanisms such as pain, loneliness, anger, and feelings of worthlessness described by participants in this study similarly reported (Culpin et al. 2022; Nduna & Sikweyiya 2015). Participants in our study expected their fathers to be present to provide emotional support and guidance; thus, their absence may have created a void, leading to feelings of loneliness and abandonment. Moreover, not knowing their father’s whereabouts could lead to confusion, frustration, or anger as they struggled to understand his absence and the reasons behind it.

Participants also reported experiencing strained psychosocial support systems because of the added burden of care on mothers and extended family members. Adolescents whose fathers had moved on to start new families reported feeling excluded from the new family and neglected by stepparents. Despite these challenges, some participants were able to find closure. This was often due to the support of the extended family members and, in some cases, when biological fathers kept in contact with their sons. In support of our findings, literature shows that in the absence of biological fathers, other male family members often step in as social fathers (Clowes et al. 2013), with grandparents sometimes playing a more important role than surviving parents (Beegle et al. 2010). While there was little mention of social fathers in our study, some participants shared the role that grandparents played in caregiving, support, and providing closure on father absence. Moreover, while extended family members can provide psychosocial support (Anderson 2015), some participants were abandoned or excluded by family members, possibly due to resource constraints. Research shows that exclusion from paternal families can disrupt social support and may lead to internalized self-stigma in children (Nduna & Jewkes 2012), which may affect youth living with HIV more seriously (Toth et al. 2018). In our findings, a participant living with HIV shared how father absence negatively affected him, as he had to rely only on his mother for HIV treatment and care support.

Our findings show that participants experienced a perceived or real loss of identity and self-esteem due to father absence. Some expressed this as a missed opportunity to learn their culture and connect with paternal families, while for others, it resulted in feelings of inadequacy or not measuring up as men. The loss of a paternal connection is linked to a broader societal expectation of masculinity, which participants perceived as an impediment to their attainment of culturally accepted masculine behavior. This could be due to the perceived lack of paternal guidance on societal expectations of manhood within cultural contexts, without which participants may be left feeling uncertain (Bitalo et al. 2024). Thus, father absence creates gaps in young men’s ability to learn behaviors and responsibilities to fulfill masculine roles.

Moreover, adolescent boys talked about what their father’s absence meant in relation to their cultural and masculine identities. This underscores the role of fathers as role models, mentors, and educators toward the formation of stable identities and a sense of self in adulthood. Similarly, research shows that fathers and families at large can play an important role in teaching culture and introducing children to it, shaping their identity, and acting as mediators in the transition to adulthood (Enderstein & Boonzaier 2015; Mkhwanazi et al. 2024). However, in South Africa’s multicultural context, masculinity ideals cannot be a monolithic concept. This is because factors such as age, culture, time, and place intersect to reveal multiple ways that young men embody or challenge hegemonic forms of masculinity (Morrell et al. 2012). As our findings revealed, some young people rejected the negative patterns associated with their fathers’ absence, such as neglect, and expressed a commitment to being present and involved fathers in their futures. This shows that our participants, as young men, are not just mere recipients of societal norms but are also actively drawn from alternative ways of understanding and expressing masculinity (Barker & Ricardo 2005).

Research indicates that father absence can lead to low self-esteem, pessimism about the future, and negative perceptions of fatherhood in young boys (Sylvester & Bojuwoye 2011). However, other studies suggest that despite these challenges and experiences, some young men still actively choose to be present fathers, rejecting the patterns of their upbringing (Enderstein & Boonzaier 2015). In some cases, father absence was linked to a diminished sense of identity for young adults, especially when they did not use their fathers’ surnames (Smith et al. 2014). In the South African context, literature shows that this could be due to maternal non-disclosure of fathers’ identities, while in some cases, fathers may not have custody or use their last name on the child without paying a price, a requirement in some cultures (Manyatshe & Nduna 2014).

We found that father absence results in perceived financial consequences, especially when the burden of care is transferred to other caregivers. In the context of South Africa, where the AIDS epidemic has left many children orphaned, the caregiver burden is intensified due to limited resources to cater for children in need of care (Kidman & Thurman 2014). In the context of South Africa, where gender roles may be inequitable, this issue is heightened by the expectation of men to provide for the family, while there are fewer opportunities for education and employment crucial for women empowerment (Ranganathan et al. 2022). Thus, the absence of men may be felt more as it leaves a significant gap with financial hardships. The employment status of fathers can impact the emotional availability of parents, as financial pressures associated with unemployment can limit available time and increase stress levels, which can affect parenting capacity (McGill 2014; Mkhwanazi et al. 2024). However, the link between employment and emotional availability may be mediated by factors such as mental health and parental styles (Diniz et al. 2023). Moreover, other scholars have also argued that the prominent view of fathers as predominantly responsible for the financial support of families can inhibit the emergence of other roles that fathers can play (Mavungu 2013).

Several participants report experiences of being raised by single parents or extended family members who struggled financially. In some cases, interest in the absent father’s identity is driven by difficult circumstances in the maternal home, such as financial hardship or social exclusion from the family (Nduna & Jewkes 2011). A study conducted in a South African township found that people with unresolved paternity identities constituted a bigger proportion of people who sought financial and psychosocial support from civic and community organizations (Nduna et al. 2011). This suggests that father absence can also carry broader, community-level implications, where resources may be required to support families facing financial hardship.

Finally, the study found that participants reacted differently to their fathers’ absence, with some participants reporting finding closure. Moreover, different circumstances, such as the age at which their father left or experiences associated with their absence, influenced how participants reached closure or acceptance of their father's absence. Most participants reported that their fathers’ absence occurred during early childhood, leading to more painful experiences when they were younger but improving with age. Other participants found closure through the support of extended family members, emphasizing the role of support systems in facilitating resilience. Research supports that the timing of absence is crucial, with paternal death between the ages of six and fifteen being especially impactful and affecting developmental and future outcomes (TenEyck et al. 2023). This period intersects with critical stages of a child’s emotional and psychological development, where children develop a stronger sense of identity and self-esteem. Similar to our findings, research also shows that communication between the father and son has a moderating effect on the effects of father absence, as it has been shown to impact life satisfaction after parental divorce (Thuen et al. 2021). In our study, participants felt more positive about their relationships with their absent fathers if they maintained communication and contact. This is likely because ongoing contact provides a sense of continuity and may reduce feelings of abandonment despite physical absence. Intervention programs in South Africa, particularly those that encourage fathers to be more involved, such as the One Man Can project by the Sonke Gender Justice organization, have proven effective in mobilizing men toward building healthier relationships (Van Den Berg et al. 2013). However, these are still limited, as most are either not specific to young men’s issues or are aimed at other issues, such as gender-based violence and HIV, such as the Brothers for Life and Men as Partners programs (Luphondo & Stroud 2012; Palitza 2011). Such programs are especially valuable when they redress the barriers preventing them from fully participating in their children’s lives (Erasmus et al. 2020).

### Strengths and Limitations

The study reports findings from young males aged 16–20, making it relevant to adolescents and offering locally grounded insights for tailored interventions. Including participants with varying HIV statuses adds depth by illustrating how father absence may intersect with other factors. However, some responses were elicited through generalized questions about the absence of family members, which may have limited the specificity of insights related to father absence. While a strength of this study is that it solicits boys’ lived experiences, this approach also carries the potential for misunderstanding or incomplete recollection of events, particularly those that occurred when they were too young to fully comprehend their fathers’ absence.

## Conclusion

Our study reports on the experiences of adolescent boys who do not reside with their biological fathers. The findings reveal that father absence significantly impacts emotional well-being, social identity, financial and psychosocial support systems, and resilience. This underscores the broader societal implications of father absence in South Africa, including the disruption of family structures and developmental challenges faced by young men. Addressing these issues requires multifaceted, targeted interventions to provide these young men with the emotional, social, and economic support needed to navigate these challenges effectively. Such interventions may include mentorship programs, cultural education initiatives, and community-based programs designed to help young men affected by father absence and equip them with healthier parenting practices for the future.

## Data Availability

The data used in this study are available upon request.
